# Structure and molecular evolution of the barcode fragment of cytochrome oxidase I (COI) in *Macrocheles* (Acari: Mesostigmata: Macrochelidae)

**DOI:** 10.1002/ece3.9553

**Published:** 2022-12-08

**Authors:** Najme Khakestani, Malihe Latifi, Esmaeil Babaeian, Wayne Knee, Samin Hosseini

**Affiliations:** ^1^ Department of Plant Protection, Faculty of Agriculture Vali‐e‐Asr University of Rafsanjan Rafsanjan Iran; ^2^ Centre for Biodiversity Genomics University of Guelph Guelph Canada; ^3^ Canadian National Collection of Insects, Arachnids, and Nematodes, Agriculture and Agri‐Food Canada Ottawa Ontario Canada

**Keywords:** Bayesian inference, divergence time, maximum likelihood, molecular dating, phylogeny

## Abstract

Consisting of approximately 320 species, *Macrocheles* is the most widely distributed genus in the family Macrochelidae. Though some studies have focused on the description of Macrochelidae using molecular techniques (e.g., RAPD) and sequencing of some genes, the interspecies relationships within *Macrocheles* still remain uncertain. As such, in the present study, we examine all publicly available data in GenBank to explore the evolutionary relationships, divergence times, and amino acid variations within *Macrocheles*. Exploring the patterns of variation in the secondary protein structure shows high levels of conservation in the second and last helices, emphasizing their involvement in the energy metabolism function of the cytochrome oxidase subunit I enzyme. According to our phylogenetic analysis, all available *Macrocheles* species are clustered in a monophyletic group. However, in the reconstructed trees, we subdivided *M. merdarius* and *M. willowae* into two well‐supported intraspecific clades that are driven by geographic separation and host specificity. We also estimate the divergence time of selected species using calibration evidence from available fossils and previous studies. Thus, we estimate that the age of the Parasitiformes is 320.4 (273.3–384.3) Mya (Permian), and the Mesostigmata is 285.1 (270.8–286.4) Mya (Carboniferous), both with likely origins in the Paleozoic era. We also estimate that *Macrocheles* diverged from other Mesostigmata mites during the Mesozoic, approximately 222.9 Mya.

## Graphical abstract

In the present study, we used all available data to explore the evolutionary relationships, divergence time, and amino acid variation of COI barcode fragment of *Macrocheles*. Our results revealed the less conserved structure of COI in *Macrocheles* species compared with arthropods. Exploring the variation pattern based on the secondary and three‐dimensional structures of proteins indicates the high level of conservation in the second and last helices emphasizing their involvement in the energy metabolism function of the COI enzyme. According to our phylogenetic analysis, all available *Macrocheles* species have been clustered in a phylogenetic group. However, due to poor sampling and lack of enough data, our phylogenetic study cannot reflect the true evolutionary relationships between *Macrocheles* species. In the reconstructed trees, M. merdarius and M. willowae were subdivided into two well‐supported intraspecific clades, which are driven by geographic separation and host specificity.

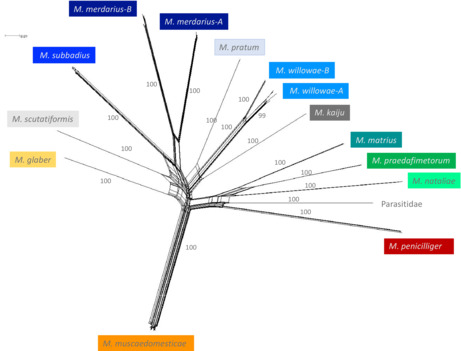

## 1 INTRODUCTION

Macrochelidae (Mesostigmata) is an ecologically and behaviorally diverse family that is represented by more than 470 described species across 21 genera (Beaulieu et al., 2011; Emberson, 2010; Krantz, 2018). Macrochelids are widespread predacious mites found in soil, leaf litter, decaying organic matter, dung, and carrion. Macrochelids typically feed on small arthropods, eggs and larvae of flies, nematodes, and oligochaetes (Gereson et al., 2003; Ito, 1970; Krantz, 1983). As predators, they influence the population growth of other micro‐invertebrates (Geden & Axtell, 1988; Perotti et al., 2001; Perotti & Bachmann, 1999) consequently, they may have effects on the advancement and composition of ephemeral micro‐ecosystems. Macrochelids are frequently associated with a diverse assemblage of insect orders (i.e., Coleoptera, Hymenoptera, Diptera, Orthoptera) (Halliday, 2000; Krantz, 1998; Mašán, 2003).

The cosmopolitan macrochelid genus, *Macrocheles*, represented by at least 320 described species, is the most diverse genus of the family (Emberson, 2010). Like many other Acari genera, *Macrocheles* has undergone several taxonomic revisions and been divided into several subgenera and species groups (Bregetova, 1977; Evans & Browning, 1956; Hyatt & Emberson, 1988; Krauss, 1970; Mašán, 2003; Walter & Krantz, 1986). The most recent and comprehensive revision based on morphological characters divided *Macrocheles* sensu lato into three genera: *Macrocheles* sensu stricto, *Nothrholaspis*, and *Macrholaspis* (Emberson, 2010).

Species of *Macrocheles* show a relatively small scale of variability in morphological features, and unknown cryptic diversity can always be uncovered using molecular techniques. Molecular techniques have proven highly effective for exploring the phylogenetic relationships and species boundaries of mites (Dabert et al., 2011; Li et al., 2010; Skoracka et al., 2012; Skoracka & Dabert, 2010; Yang et al., 2011; Zhao et al., 2014, 2015). However, few studies have used molecular information to explore the phylogenetic relationships of macrochelids. Recent studies on the species of *Macrocheles* focused mainly on the descriptions of new species (Knee, 2017; Niogret et al., 2007; Niogret & Nicot, 2008). The interspecies relationships within *Macrocheles*, as well as the time of divergence from its most recent common ancestors, remain uncertain.

Mites have a long evolutionary history, dating back to at least 410 million years ago (Mya) based on their presence in early terrestrial fossil layers (Dabert et al., 2010; Dubinin, 1962; Hirst, 1923; Xue et al., 2017). However, their evolutionary history is only partially understood, and mites remain one of the least studied major branches of the animal tree of life (Dunlop & Alberti, 2008; Krantz & Walter, 2009). Mites are poorly represented in the fossil record compared with other arthropods, which may be due to mites being difficult to preserve, quite small, and easily overlooked in fossils (Sidorchuk, 2018). Of the two Acari superorders, the Acariformes are far better represented in the fossil record than the Parasitiformes (Fuente, 2003). Acariformes first originated c. 435 MYA in the Llandovery Silurian (Dabert et al., 2010) and have been collected across the Cretaceous (Arillo et al., 2022; Porta et al., 2020; Sidorchuk et al., 2015). The oldest parasitiform fossils are from 90 Mya. (Dunlop et al., 2014; Klompen & Grimaldi, 2001). The poor representation of Parasitiformes in the fossil record could be because parasitiform mites rarely colonize the bark of trees and thus are rarely captured in amber and fossilized (Dunlop et al., 2014; Van der Hammen, 1977). The Parasitiformes are likely much older than 90 Mya (Dunlop & Bernardi, 2014), but the poor representation of these mites in the fossil record impedes our understanding of their evolutionary origins. Molecular dating provides a means to uncover the origins of these mites.

In the present study, we aim to: (i) explore the phylogenetic relationships of *Macrocheles* species using the barcode region of cytochrome oxidase subunit I (COI) from publicly available data on GenBank, (ii) estimate the time of divergence for the genus using a molecular clock and available COI data, and (iii) characterize the amino acid and protein structure of COI in *Macrocheles*.

## 2 METHODS

### 2.1 Multiple sequence alignment and phylogenetic analysis

We began by downloading from GenBank all publicly available nucleotide sequences spanning the barcode region of COI, for named *Macrocheles* species (10 June 2021) (Table A1). After aligning the nucleotide sequences of these species using MAFFTv.7 (Katoh & Standley, 2013), we visualized and inspected them manually using Mesquite v.3.61 (Maddison & Maddison, 2015). We selected a Parasitidae species (MN353534) as the outgroup because, based on BLAST search results, its sequence was one of the most closely related to Macrochelidae.

Implementing the ModelFinder program of IQ‐TREE, we generated a maximum likelihood (ML) based on nucleotide sequences of selected taxa (Martin et al., 2015), creating an ML tree based on the best substitution model. To evaluate branch support for the ML tree, we used 1000 replicates of Ultrafast bootstrap (UFBoot) (Felsenstein, 1985).

Furthermore, we performed Bayesian analysis using the Linux version of MrBayes 3.2.7 (Ronquist et al., 2003) to reconstruct a Bayesian inference (BI) tree under mixed models with four MCMCs, two independent runs, and 10^7^ generations, sampling every 10,000th generation and burn‐in proportion of 0.25. For validation of the MrBayes result, we used Tracer 1.7.1 (http://tree.bio.ed.ac.uk/software/tracer/) to determine whether all parameters were well mixed and converged, calculating the effective sample size (ESS) values of parameters per separate runs. A calculated ESS value of a parameter less than 100 indicates poor mixing, which represents low efficiency of sampling of parameters using MCMC algorithms; thus we improved the ESS value by running the MCMC for a longer time. We then visualized the created IQ‐TREE and the reconstructed consensus tree using MrBayes 3.2.7 based on 50% majority rules using FigTree v1.4.3 (http://tree.bio.ed.ac.uk/software/figtree/). To compare the congruency of topology among the two phylogenetic trees using the IQ‐TREE program, we employed the following RELL method (Kishino et al., 1990), one‐sided Kishino–Hasegawa (KH) test (Kishino & Hasegawa, 1989), Shimodaira–Hasegawa (SH) test (Shimodaira & Hasegawa, 1999), weighted KH and SH tests, expected likelihood weight (Strimmer & Rambaut, 2002), and approximately unbiased tests (Shimodaira, 2002) with 10,000 RELL replicates.

Finally, we examined the phylogenetic relationships of taxa using SplitsTree v4.16.1 (Huson & Bryant, 2006) by creating a Neighbor‐Net network from uncorrected patristic distances with 1000 bootstrap replications using the default setting.

### 2.2 COI properties and protein structure

To examine the protein structure of COI among the 12 available species, we selected one sequence per species that did not have any ambiguous bases for further analysis. We calculated the percent identity of amino acid sequences of 12 selected species using BLAST's blastp version. To identify how many characters of target and query taxa were identical. We also calculated the pairwise distance between amino acid sequences by MEGAX using the default setting (Kumar et al., 2018).

After aligning the amino acid sequences of selected species using MAFFTv.7 (Katoh & Standley, 2013), we used different tools to examine the structure of the proteins at three levels: primary, secondary, and tertiary. To examine the primary structure of selected proteins, we counted the frequency of amino acids per protein and visualized the generated multiple sequence alignment (MSA) using a web‐based sequence manipulation suite program to see the conservation pattern among the taxa (Stothard, 2000). (http://www.bioinformatics.org/SMS/multi_align.html). We detected the position of the functional domain of each protein by searching each one in the Pfam database (https://pfam.xfam.org/).

To estimate the position of helices in the secondary structure of the proteins, we used TMHMM v2.0 (http://www.cbs.dtu.dk/services/TMHMM/) and ExPASy‐ProtScale (https://web.expasy.org/protscale/; Gasteiger et al., 2005) servers. The TMHMM estimated the probability and position of transmembrane helices, while the ExPASy‐ProtScale analyzed the amino acid secondary structure by generating the hydropathy graphs.

To compare the tertiary structure of selected taxa, we first created a 3D model of *Macrocheles willowae*, which has the longest length among the 12 selected sequences. We validated the model using ProSA‐web (Wiederstein & Sippl, 2007) and a Ramachandran plot (https://zlab.umassmed.edu/bu/rama/index.pl). To illustrate the conservation pattern of all sequences in relation to the 3D structure, we mapped the 12 selected amino acid sequences on the surface of the modeled structure using the ConSurf server (Ashkenazy et al., 2016).

Finally, we detected the physicochemical properties of the proteins using the ProtParam program (https://web.expasy.org/cgi‐bin/protparam).

### 2.3 Molecular dating

The estimated age of selected *Macrocheles* species was determined using BEASTv2.6.3. To accurately calibrate the estimated age analysis, however, reliable fossils would be required. Although extant parasitiform mites occur in a wide array of habitats worldwide, they are infrequently collected in fossil records. Acariformes mites make up most fossilized mite collections (Dunlop et al., 2018). As a result, the fossil records for Mesostigmata in our analysis were limited to just *Aclerogamasus* sp. (Parasitidae) and *Myrmozercon* sp. (Laelapidae). Therefore, we used the estimated age of Parasitiformes, which is based on the latest Acari dating study of Arribas et al. (2020), as the secondary calibration (Table 1). This calibration method is commonly used to calibrate the trees in a new study based on an estimated date of a node from previous studies. To calculate the uncertainty of estimates, we used lognormal distribution and normal distribution for the fossil and secondary calibrations, respectively.

**TABLE 1:** Calibration points and their prior setting used for molecular dating in the present study.

Node^a^	Fossil/secondary calibration		Reference
	Age (mean‐Mya)	Prior	
Mes	279^b^	Normal (mean:279, sd:4)	Arribas et al. (2020)
Lae	44^b^	Lognormal (offset:44, mean: 20, sd: 1 in real space)	Witlanski (2000)
Par	44^b^	Lognormal (offset:44, mean: 20, sd: 1 in real space)	Dunlop and Selden (2009)

^a^ The node Mes, Lea, and Par are related to the crown node of clades Mesostigmata and Laelapidae and Parasitidae.

^b^ Are related to secondary and fossil calibration, respectively.

For molecular dating analysis, we employed the GTR + G4 substitution model (suggested by the IQ‐TREE analysis) and the Yule process model, along with a relaxed lognormal clock as the simplest speciation model. We set the screen log to 10^4^ and the chain length to 100 million, and we used the configuration file generated by BEAUTYv2.6.3. To check the congruency among parameters, we evaluated the results by Tracer v1.6 (Bouckaert et al., 2014).

Finally, we summarized the tree information produced by BEAST using TreeAnnotator v2.6.3 (implemented in BEAST tools package) with a burn‐in of 10% and then displayed it with FigTree v.1.4.4.

## 3 RESULTS

### 3.1 Phylogenetic analysis

The COI alignment generated based on nucleotide sequence from publicly available GenBank data on 111 *Macrocheles* specimens and one Parasitidae species produced an alignment with 658 characters in total, with 45% parsimony‐informative, 3% singleton, and 52% constant sites. The best fit model based on the Bayesian information criterion was TIM2+F+I+ by the ModelFinder program implemented in IQ‐TREE. The congruency of two BI and ML trees that we examined using several tests implemented in IQ‐TREE program are summarized in Table 2. While the log‐likelihood of the two trees was identical (marked as a plus) based on the calculated bootstrap portion of both RELL (Kishino et al., 1990) and expected likelihood weight (Strimmer & Rambaut, 2002) methods, the estimated *p*‐values of the remaining tests (Table 2) were less than 0.05 indicating the significant difference between the two trees (marked as a minus).

**TABLE 2:** The result of different topological congruency tests performed by IQ‐TREE program^a^.

Trees	logL	deltaL	Bp‐RELL	p‐KH	p‐SH	p‐WKH	p‐WSH	c‐ELW	p‐AU
ML	5396.41	0	0.509 +	0 −	0 −	0 −	0 −	0.5 +	0 −
BI	5396.41	0	0.491 +	0 −	0 −	0 −	0 −	0.5 +	0 −

^a^ logL is log of likelihood; deltaL is the difference of calculated logL between two trees; bp‐RELL is bootstrap proportion using RELL method (Kishino et al., 1990); p‐KH is the estimates p‐value of the one‐sided Kishino–Hasegawa test (Kishino & Hasegawa, 1989); p‐SH is the p‐value of the Shimodaira–Hasegawa test (Shimodaira & Hasegawa, 1999); p‐WKH is the p‐value of the weighted Kishino–Hasegawa test; p‐WSH is the p‐value of the weighted Shimodaira–Hasegawa test; c‐ELW is expected likelihood weight (Strimmer & Rambaut, 2002), and p‐AU is the p‐value of approximately the unbiased (AU) test (Shimodaira, 2002); Plus signs indicate 95% confidence test. Minus sign denotes significant exclusion.

**FIGURE 1:**
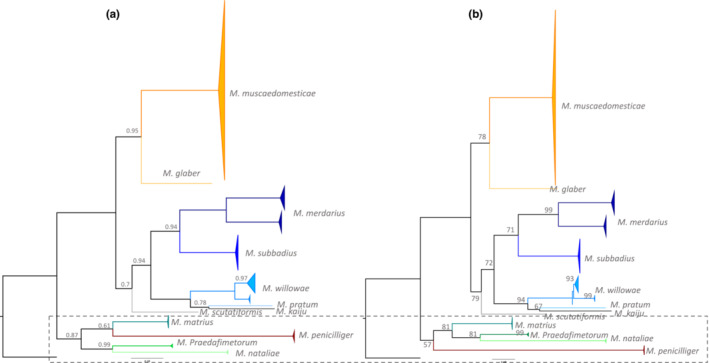
(a) BI phylogenetic tree created by MrBayes and (b) the reconstructed ML tree generated by IQ‐TREE. The phylogenetic analysis was based on nucleotide sequences including 658 characters of COI marker of 111 *Macrocheles* mites representing 12 species rooted by a Parasitidae species. The estimated posterior probability of less than 1 and bootstrap support value of less than 100 of the main nodes are shown for each node in a and b, respectively. The trees have been illustrated using Figtree, and each species is denoted by a unique color. The clade with different clustering patterns between two trees has been shown by a dashed box.

All 12 morphologically defined *Macrocheles* species were well‐supported in both the reconstructed BI and ML trees, occurring in distinct clades with high bootstrap support (>99) and high posterior probability (1.0). Incongruences between the two trees (the clades in a dashed box in Figure 1) were found in four clades, where *M. penicilliger* in the ML tree diverged from *M. matrius*, *M. praedafimetorum*, and *M. nataliae*, in the BI tree, which is the sister group of *M. matrius*. We further divided two species, *M. merdarius* and *M. willowae*, into two well‐supported clades.

The reticulated network analysis demonstrated that the clustering pattern of the 12 examined macrochelid species was generally congruent with the ML and BI trees.

**FIGURE 2:**
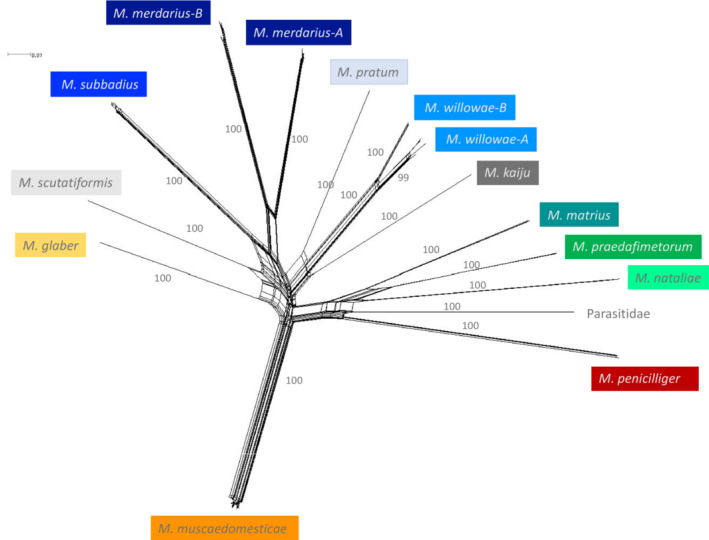
Network analysis based on the nucleotide sequence of a 658 fragment of COI from 111 *Macrocheles* specimens and 12 species.

Most of the morphologically defined species were well delineated by the network analysis, while the relatively large intraspecific divergences among *M. merdarius* and *M. willowae* were also well‐supported (Figure 2).

### 3.2 COI protein structure and properties

**FIGURE 3:**
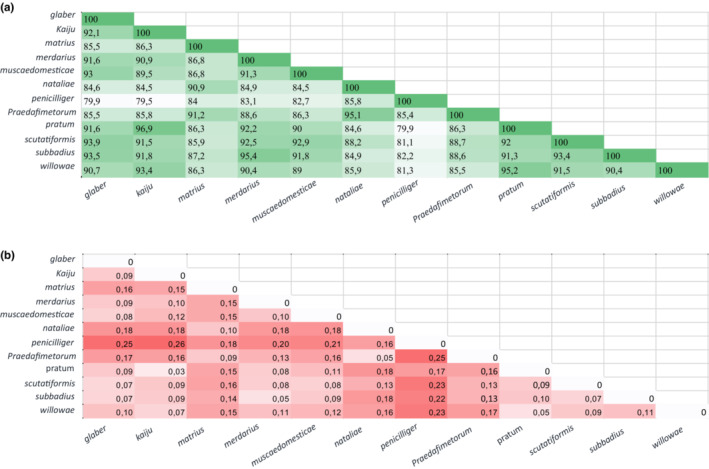
Heatmap based on a matrix including the (a) identity (%) between the amino acid sequences of 12 *Macrocheles* species, calculated using blastp, and (b) pairwise distance between amino acid sequence of 12 species calculated by MEGA X. The color intensity indicates the level of identity and distance between the species. The white color indicates the lowest number while dark green, and dark red color represents the highest number in two created heatmaps.

The amino acid sequences of the 12 taxa used in this study are 79.4%–96.9% identical. The pairwise distance between taxa varied from 0.03 to 0.25. Among the 12 species, *M. pratum* and *M. kaiju* were the most similar in amino acid composition, while *M*. *penicilliger* and *M. kaiju* were the least similar in amino acid composition (Figure 3).

We have summarized several physicochemical properties of selected proteins in Table 3. The highest value per each property is indicated as a bold number.

**TABLE 3:** Physicochemical properties of the barcode fragment of COI from *Macrocheles* based on amino acid sequences of 12 machrochelid species.

	Name	Instability index	Extension coefficient	PI	Length	Molecular weight
1	*M. glaber*	32.38	31,970	4.96	214	23254.74
2	*M. kaiju*	36.53	**38,960**	5.67	226	24754.53
3	*M. matrius*	38.88	33,460	5.30	219	24129.97
4	*M. merdarius*	32.10	33,460	5.30	219	23952.54
5	*M. muscaedomesticae*	35.86	33,460	5.64	219	24075.68
6	*M. nataliae*	37.89	**38,960**	5.51	227	25113.73
7	*M. subbadius*	33.67	33,460	5.30	219	23982.51
8	*M. penicilliger*	33.88	34,950	4.78	219	24145.80
9	*M. praedafimetorum*	**39.65**	**38,960**	5.58	227	25054.74
10	*M. pratum*	34.13	**38,960**	**6.29**	**230**	**25217.01**
11	*M. scutatiformis*	35.08	31,970	5.34	212	23063.45
12	*M. willowae*	34.79	**38,960**	6.02	**230**	25379.18

*Note*: The highest value per each property is indicated as a bold number.

The estimated instability index of all proteins varied between 32.10 (for *M. merdarius*) to 39.65 (for *M. praedafimetorum*). The instability index of all selected proteins is less than 40, indicating that thy are stable proteins in vitro (Ignatova et al., 2007). By contrast, the grand average of hydropathicity of the selected 12 proteins (sum of the hydropathicity = value of each amino acid / total number of amino acids) ranged from 0.731 to 0.931. This is related to *M. penicilliger* and *M. praedafimetorum*, which can be considered the most and least hydrophobic proteins, respectively.

**FIGURE 4:**
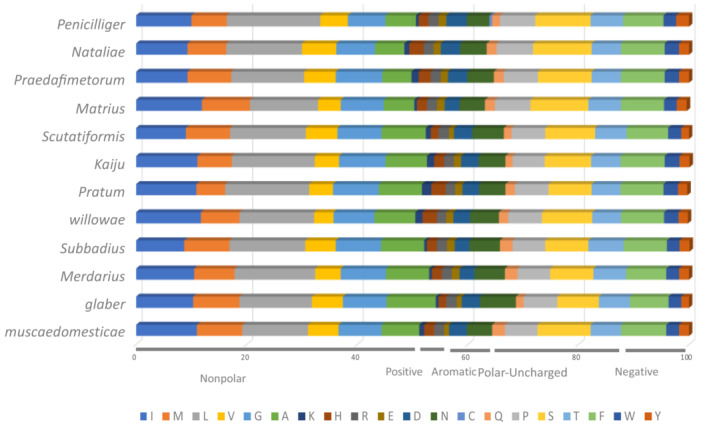
The frequencies of amino acid in the barcode region of COI for 12 *Macrocheles* species. The amino acids have been clustered based on biochemical properties into five groups named, nonpolar, positive, aromatic, polar‐unchanged, and negative, which is shown below the generated graph.

The frequencies of amino acids in COI for the 12 *Macrocheles* species were generally quite similar (Figure 4).

**FIGURE 5:**
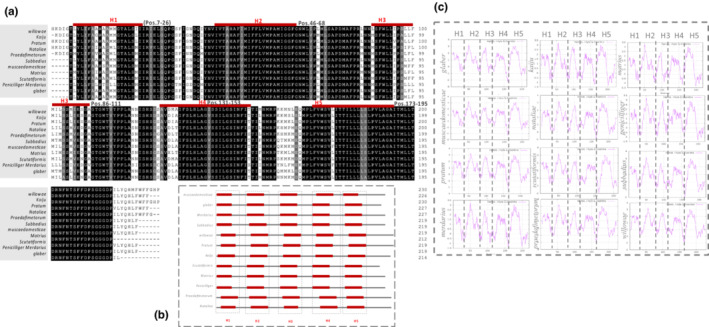
(a) The conservation pattern of amino acid sequences of 12 *Macrocheles* species. The position of each transmembrane helices has been indicated by a red bar. The start and end position of each helix has been shown next to the bar. The black columns are the characters, which are conserved over 12 species, and (b) the schematic presentation of the secondary structure of 12 *Macrocheles* species. Each red bar represents the position of transmembrane helices based on the result of TMHMM and pfam (Tables A2 and A3). (c) the hydropathy profiles estimated by ProtScale program. The y‐axis is related to the hydrophobicity score (higher score, more hydrophobicity), and x‐axis shows the position of amino acids per taxa.

The secondary structure of the COI region contains five helices (H1–H5) connected by five loops. Of the 230 amino acid characters we examined, 67% were conserved across all 12 *Macrocheles* species, 36% of which were located in helix regions (Figure 5a; Tables A2 and A3). The predicted positions of each helix were quite similar across species, with only minor variability (Figure 5c). Across the 12 species, 50, 91, 35, 78, and 87% of amino acid sequences located in H1, H2, H3, H4, and H5 regions, respectively, were conserved. Accordingly, H2 is the most conserved helix, followed by H5. The hydropathicity profile in all species estimated by the ProtScale program (Figure 5c) was quite similar. As is clear from the graphs in Figure 5c, each of the five helices (H1–H5) has a distinctly high hydrophobicity score.

**FIGURE 6:**
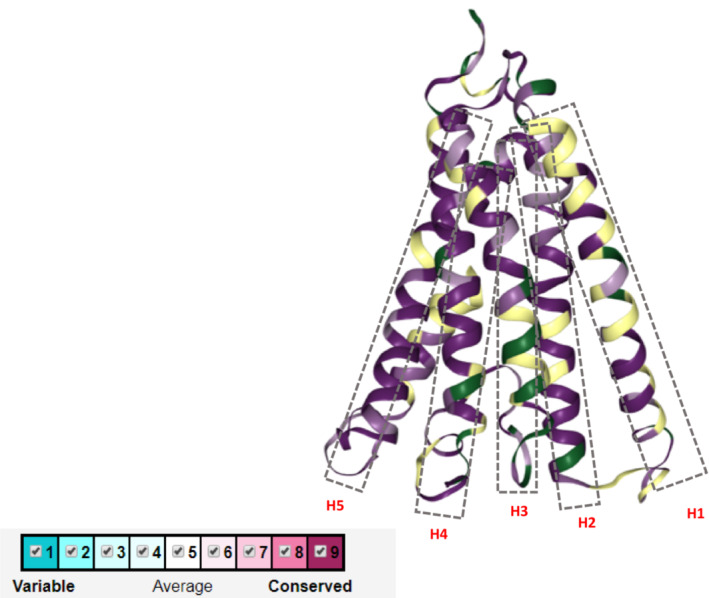
The conservation pattern of 12 amino acid structures on a modeled 3D structure of *M. willowae*.

**FIGURE A1:**
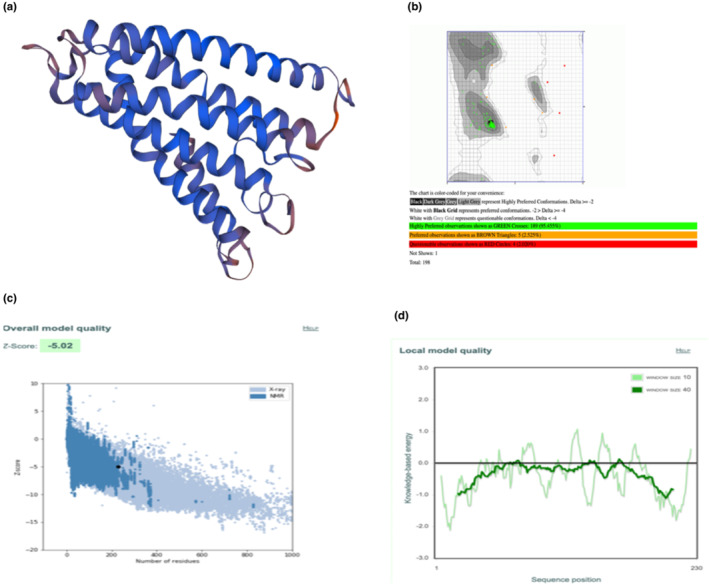
(a) Three‐dimensional structure orientation of COI marker of *M. willowae* (AUC63267) predicted by SWISS MODEL online tools (http://swissmodel.expasy.org/) based on an available COX protein (7JRO.1) (Maldonado et al., 2021) in public protein data bank (PDB) as model. 3D structure evaluation of the model has been displayed by (b) Ramachandran plot and (c and d) ProsA‐web tool. In graph c, the x‐axis is related to z‐score (indicating the quality of 3D structure) and y‐axis indicates the number of available 3D structure, which are determined by different methods; the light blue and dark blue dots shows the z‐score of the proteins, which are the result of X‐ray or NMR determination methods. The z‐score of our model have been shown by a black dot in graph (c). The dark and light green lines in graph (d) display estimated average energy over each 40‐ and 10‐ residue fragment, respectively.

After analyzing the primary and secondary structures of the studied proteins, we mapped all amino acid sequences on the predicted model of the COI marker, *M. willowae* (AUC63267) (Figure A1). The modeled 3D structure confirmed a high conservation of COI amino acid sequences in the H2 and H5 regions (Figure 6).

### 3.3 *Macrocheles* divergence time

**FIGURE 7:**
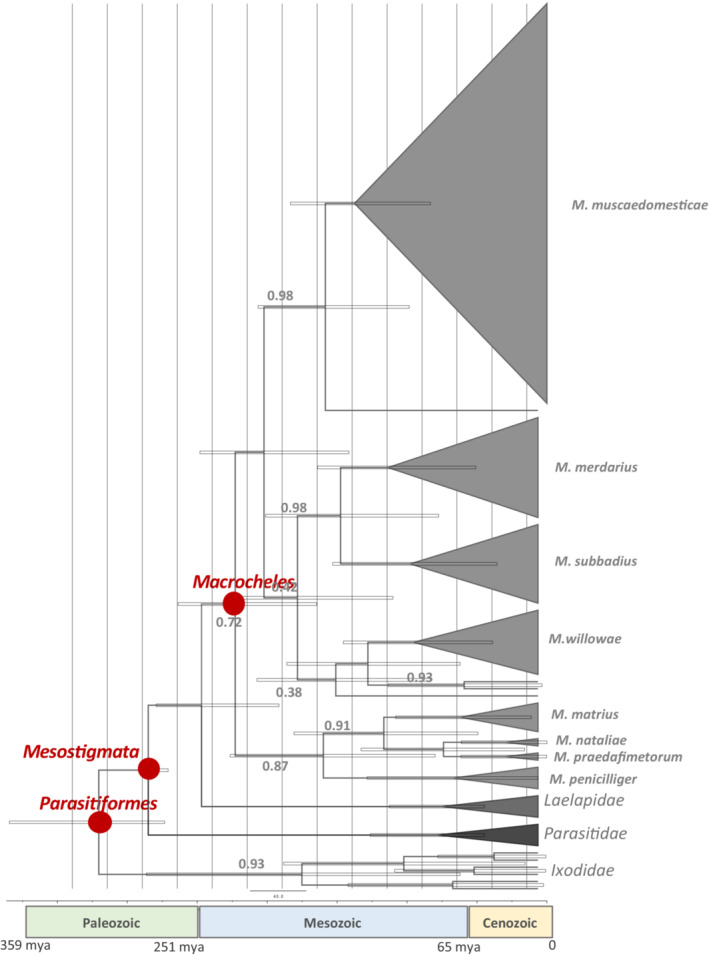
The time‐calibrated phylogenetic tree based on the nucleotide sequence of 111 *Macrocheles* species belongs to 12 species and 14 species belongs to Laelapidae (*Hypoaspis*: JX835261; Androlaelaps *casalis*: MH983653; *Stratiolaelaps scimitus*: MH983671; *Gaeolaelaps aculeifer*: MH983856), Parasitidae (*Parasitus hyalinus*: MH983855; Gamasodes *spiniger*: MH983603; *Parasitellus fucorum*: MW367969; *Pergamasus misellus*: MN348250); and Ixodidae (*Ixodes scapularis*: MN357814; *I. pavlovskyi*: AB231669; *Hyalomma marginatum*: KX000636; *H. lusitanicum*: EU827710; *Amblyomma triguttatum*: MN106719; *A. variegatum*: GU062743). Horizontal bars represent 95% highest posterior density (HPD) around mean node ages. The crown node of Macrocheles, Mesostigmata, and Parasitiformes have been indicated as the red node in the tree. The estimated posterior probability has been written each node. The bars at the bottom of the tree indicate the geological time scale. The estimated posterior probability of less than 1 of the main nodes is shown for each node in the tree.

The time‐calibrated phylogeny tree created from our BEAST analysis is displayed in Figure 7 and the divergence time of the *Macrocheles* species, and other taxa used in this study are summarized in Table 4. Considering this, we estimated that the age of the Parasitiformes is 320.4 (273.3–384.3) Mya (Permian), and the Mesostigmata is 285.1 (270.8–286.4) Mya (Carboniferous), both in the Paleozoic era. However, the divergence time of *Macrocheles* from other Mesostigmata occurred in the Mesozoic, approximately 222.9 Mya. Based on nucleotide sequencing of COI, the youngest *Macrocheles* species is *M. nataliae* (29.2 Mya) followed by *M. praedafimetorum* (29.5 Mya), and the oldest is *M. muscaedomesticae* (137.6 Mya).

**TABLE 4:** The estimated time of divergence for *Macrocheles* species and higher taxa.

Name of clade	Mean age (mya)	95% HPD^a^ (Mya)
Parasitiformes	320.4	273.3–384.3
Mesostigmata	285.1	270–286.4
*Macrocheles*	222.9	164.6–263.7
*M. muscaedomesticae*	137.6	83.3–183.5
*M. subbadius*	97.8	35.7–153.2
*M. matrius*	61.9	11.2–108.2
*M. praedafimetorum*	29.5	0.2–61.5
Laelapidae	74.8	44.6–112.6
Ixodidae	175.2	61.9–286.3
*M. merdarius*	113.5	50.9–164
*M. willowae*	95.3	38.8–145.5
*M. nataliae*	29.2	0.02–61.5
*M. penicilliger*	67	6.6–128.7
Parasitidae	78	44.4–126.3

^a^ HPD: 95% highest posterior density.

## 4 DISCUSSION

In this study, we explored the phylogenetics of *Macrocheles* species using COI and characterized the amino acid and protein structure of COI for the genus using publicly available sequence data. While a few studies have focused on the molecular characterization of COI in some arthropods, there is no existing information about the COI structure of *Macrocheles*.

To gain a better understanding about the variability of COI, we compared the primary, secondary, and tertiary protein structures of COI in 12 macrochelid species. Most amino acids in the primary structure of the studied species were nonpolar; *M. glaber* had the highest nonpolar amino acid content, and *M. nataliae* had the lowest. Lysine (K) is a positive amino acid and occurred less frequently than the other amino acids in the species studied. The other two positive (histidine and arginine) and the three negative (phenylalanine, tryptophan, and tyrosine) amino acids were conserved across the species studied. Cysteine was not present in any of the taxa studied. Researchers have reported seeing a relatively infrequent occurrence of cysteine, lysine, glutamine, tyrosine, and glutamic acid in *Macrocheles* in other groups of arthropods as well (Pentinsaari et al., 2016; Sabir et al., 2019).

In the secondary and tertiary structure of the COI barcode of *Macrocheles*, helices are more variable than loops, which might be because of the role of loops in stability of the proteins (Ulmschneider et al., 2005). However, of five detected helices, the second helix and the last helix were the most conserved. This may be due to their important function of penetrating the mitochondrial membrane, which changed the structure of the enzyme and likely reduced the energy metabolism of the cells (Mathews et al., 2013).

Furthermore, we studied the phylogenetic relationship among available COI sequences of macrochelids in public datasets. The BI and ML phylogenetic analyses of the available COI data were largely congruent, and the morphologically defined species were well‐supported by the molecular data, with species separated into well‐supported monophyletic clades. *M. merdarius* and *M. willowae* were subdivided into two well‐supported intraspecific clades. The two clades of *M. merdarius* could be due to geographic separation, since specimens from Israel are found exclusively in one clade, and specimens from Spain and Poland were exclusively found in the other. By contrast, the two clades of *M. willowae* may be driven by host specificity, since specimens from one clade were collected from *Nicrophorus orbicollis* (Silphidae) and *N. carolinus*, while specimens from the other clade were collected from *N. defodiens*. Although the relationship of some species clades was not resolved based on estimated bootstrap and posterior probability, all species were clustered in a monophyletic group. Incongruencies between the two phylogenetic trees may be related to the small sample size or complexity of the evolutionary relationships among taxa. Future work should include more species and additional molecular markers to improve estimates of the true phylogenetic relationships among members of this genus.

Considering any incongruencies, we estimate that the age of the Parasitiformes is 320.4 (273.3–384.3) Mya (Permian), and the Mesostigmata is 285.1 (270.8–286.4) Mya (Carboniferous), both in the Paleozoic era. Our dating estimates are similar to those obtained by Arribas et al. (2019): Parasitiformes 310 (268–359) Mya and Mesostigmata 279 (238–329) Mya.

Arthropods are believed to have originated during the Cambrian of the early Paleozoic (Arribas et al., 2020), and some arachnid groups (e.g., spiders, scorpions, mites) diversified more heavily during the late Paleozoic (Jeyaprakash & Hoy, 2009). Arribas et al. (2020) dated the ancestor of Arthropoda back to the Cambrian period of the Paleozoic era and placed Parasitiformes, in the late Carboniferous, just over 300 Mya.

The divergence time of *Macrocheles* from other Mesostigmata occurred in the Mesozoic, approximately 222.9 Mya. During the Mesozoic, the climate warmed approximately 6–9°C warmer than the present (Sellwood & Valdes, 2006). This warming spurred the diversification of the angiosperms, which in turn resulted in an explosion of insect and mite species (Labandeira et al., 2007: Schuettpelz & Pryer, 2009).

## AUTHOR CONTRIBUTIONS

**Najme khakestani:** Writing – original draft (equal). **Malihe Latifi:** Supervision (equal). **Esmaeil Babaeian:** Writing – original draft (equal). **Wayne Knee:** Writing – review and editing (equal). **Samin Hosseini:** Methodology and data analysis (equal); writing – review and editing (equal).

## Data Availability

The data that supports the findings of this study are available in the supplementary material of this article

## APPENDIX A

**TABLE A1:** GenBank accession number and country of origin for *Macrocheles* used in this study

Name	Accession no	Country
*M. glaber*	MN351659^a^	Canada
*M. kaiju*	MF192750^a^	USA
*M. matrius*	MH983565^a^	Poland
	MH983572	Poland
	MH983736	Poland
	MH983739	Poland
	MH983849	Poland
*M. merdarius*	MH983598	Israel
	MH983619	Israel
	MH983627	Israel
	MH983633	Israel
	MH983662	Spain
	MH983664	Spain
	MH983681	Israel
	MH983707	Poland
	MH983748	Spain
	MH983777	Spain
	MH983789^a^	Poland
	MH983792	Spain
	MH983808	Israel
	MH983818	Israel
	MH983847	Spain
*M. muscaedomesticae*	MG407799	Canada
	MG407932	Canada
	MG407954	Canada
	MG408719	Canada
	MG409525	Canada
	MG410593	Canada
	MG411727	Canada
	MG412070	Canada
	MG412125	Canada
	MG412549	Canada
	MG412840^a^	Canada
	MG413424	Canada
	MG413557	Canada
	MG413889	Canada
	MG413897	Canada
M. muscaedomesticae	MG414124	Canada
	MG414294	Canada
	MG414508	Canada
	MG414858	Canada
	MG415829	Canada
	MG415832	Canada
	MG415869	Canada
	MG415884	Canada

	MG416000	Canada
	MG416003	Canada
	MG416119	Canada
	MG416528	Canada
	MG416693	Canada
	MG416748	Canada
	MG416809	Canada
	MG416824	Canada
	MH507145	Canada
	MH507146	Canada
	MH983612	Spain
	MH983613	Belgium
	MH983624	Belgium
	MH983637	Belgium
	MH983649	Belgium
	MH983656	Spain
	MH983683	France
	MH983700	Spain
	MH983705	Spain
	MH983708	France
	MH983710	Belgium
	MH983718	Belgium
	MH983726	Spain
	MH983745	Spain
	MH983752	Spain
	MH983754	Spain
	MH983759	Spain
	MH983780	Belgium
MH983799	Spain
*M. muscaedomesticae*	MH983815	France
	MH983819	France
	MN361263	Canada
*M.nataliae*	MF192752^a^	Germany
	MF192753	Germany
*M.penicilliger*	MH983699	Israel
	MH983630	Belgium
	MH983673^a^	Belgium
	MH983678	Belgium
	MH983713	Belgium
*M praedafimetorum*	MN349220	Canada
	MF192754^a^	Island
*M. pratum*	MF192751^a^	Canada
*M. scutatiformis*	MH983584^a^	Spain
*M. subbadius*		
	MG413458	Canada
	MG410112^a^	Canada
	MG410902	Canada
	MG411540	Canada
	MG412493	Canada
	MG412968	Canada
	MG414975	Canada
	MG415493	Canada
	MG415712	Canada
	MG415806	Canada
	MG416145	Canada
	MG417018	Canada
*M. willowae*	MF192749	Canada
	MF192743	USA
	MF192744	Canada
	MF192745	Canada
	MF192746	Canada
	MF192747	Canada
	MF192748^a^	Canada
	MN350805	Canada
	MN357791	Canada
	MN363456	Canada

^a^ The selected taxa as the representative of species in the present study.

**TABLE A2:** The result of Pfam indicating the start and end position of found COX1 domain and their probability (*E*‐value) per *Macrocheles* species

No.	Name	Domain	Alignment		*E*‐value
			Start	End	
1	*M. glaber*	COX1	2	214	3.1 e‐54
2	*M. kaiju*	COX1	2	226	5.0 e‐61
3	*M. matrius*	COX1	2	219	9.8 e‐56
4	*M. merdarius*	COX1	2	219	8.8 e‐57
5	*M. muscaedomesticae*	COX1	2	219	1.3 e‐56
6	*M. nataliae*	COX1	3	227	6.3 e‐63
7	*M. penicilliger*	COX1	2	219	3.6 e‐56
8	*M. praedafimetorum*	COX1	2	227	3.7 e‐64
9	*M. pratum*	COX1	3	230	1.2 e‐65
10	*M. subbadius*	COX1	2	219	6.3 e‐57
11	*M. scutatiformis*	COX1	2	210	6.4 e‐55
12	*M. willowae*	COX1	3	230	3.2 e‐64

**TABLE A3:** Results of transmembrane helices prediction of *Macrocheles* species using TMHMM server v.2.0

No.	Name	TMHs	PosH1	PosH2	PosH3	PosH4	PosH5
1	*M*. *muscaedomesticae*	5	2–21 (19)	41–63 (22)	84–106 (22)	131–153 (22)	166–185 (19)
2	*M*. *glaber*	5	2–21 (19)	41–63 (22)	84–106 (22)	126–148 (22)	168–190 (22)
3	*M*. *merdarius*	5	2–21 (19)	41–63 (22)	84–106 (22)	126–148 (22)	168–190 (22)
4	*M*. *subbadius*	5	2–21 (19)	44–63 (22)	84–106 (22)	126–148 (22)	168–190 (22)
5	*M*. *willowae*	5	7–26 (19)	46–68 (22)	89–111 (22)	131–153 (22)	173–195 (22)
6	*M*. *pratum*	5	7–26 (19)	46–68 (22)	88–110 (22)	130–152 (22)	173–195 (22)
7	*M*. *kaiju*	5	3–25 (22)	45–67 (22)	88–110 (22)	130–152 (22)	172–194 (22)
8	*M*. *scutatiformis*	5	2–21 (19)	41–63 (22)	84–106 (22)	126–148 (22)	168–190 (22)
9	*M*. *matrius*	5	2–21 (19)	41–63 (22)	84–106 (22)	126–148 (22)	168–190 (22)
10	*M*. *penicilliger*	5	2–21 (19)	41–63 (22)	84–106 (22)	126–148 (22)	168–190 (22)
11	*M*. *praedafimetorum*	5	7–29 (22)	44–66 (22)	87–106 (19)	135–157 (22)	170–192 (22)
12	*M*. *nataliae*	5	7–26 (19)	46–68 (22)	89–107 (18)	136–158 (22)	171–193 (22)

*Note*: TMHs is the abbreviation of number of predicted transmembrane. PosH1‐PosH5 indicates the nucleotide position of each estimated TM, and the number in parenthesis is related to the length of each helix.
